# Process failure mode and effects analysis for external beam radiotherapy: Introducing a literature-based template and a novel action priority

**DOI:** 10.1016/j.zemedi.2024.02.002

**Published:** 2024-02-29

**Authors:** Dominik Kornek, Christoph Bert

**Affiliations:** Department of Radiation Oncology, Universitätsklinikum Erlangen, Friedrich-Alexander-Universität Erlangen-Nürnberg (FAU), 91054 Erlangen, Germany; Comprehensive Cancer Center Erlangen-EMN (CCC ER-EMN), 91054 Erlangen, Germany

**Keywords:** Prospective risk assessment, Process failure mode and effects analysis (PFMEA), Template, External beam radiation therapy (EBRT), Action priority (AP)

## Abstract

**Purpose:**

The first aim of the study was to create a general template for analyzing potential failures in external beam radiotherapy, EBRT, using the process failure mode and effects analysis (PFMEA). The second aim was to modify the action priority (AP), a novel prioritization method originally introduced by the Automotive Industry Action Group (AIAG), to work with different severity, occurrence, and detection rating systems used in radiation oncology.

**Methods and materials:**

The AIAG PFMEA approach was employed in combination with an extensive literature survey to develop the EBRT-PFMEA template. Subsets of high-risk failure modes found through the literature survey were added to the template where applicable. Our modified AP for radiation oncology (RO AP) was defined using a weighted sum of severity, occurrence, and detectability. Then, Monte Carlo simulations were conducted to compare the original AIAG AP, the RO AP, and the risk priority number (RPN). The results of the simulations were used to determine the number of additional corrective actions per failure mode and to parametrize the RO AP to our department’s rating system.

**Results:**

An EBRT-PFMEA template comprising 75 high-risk failure modes could be compiled. The AIAG AP required 1.7 additional corrective actions per failure mode, while the RO AP ranged from 1.3 to 3.5, and the RPN required 3.6. The RO AP could be parametrized so that it suited our rating system and evaluated severity, occurrence, and detection ratings equally to the AIAG AP.

**Conclusions:**

An adjustable EBRT-PFMEA template is provided which can be used as a practical starting point for creating institution-specific templates. Moreover, the RO AP introduces transparent action levels that can be adapted to any rating system.

## 1 Introduction

Process failure mode and effects analysis (PFMEA) is a systematic method for identifying and managing failure chains. To this end, experienced professionals study processes and their potential failures, independent of their actual occurrence [1]. As an easy-to-implement and cost-efficient method, PFMEA has been widely adopted across radiotherapy departments, e. g., in the U. S. [2], Brazil [3], Germany [4], Italy [5] and Spain [6].

However, practical challenges somewhat impede the acceptance of risk assessments. For instance, a recent German survey disclosed national deficits in risk management knowledge: 80% of the responding institutions evaluated their knowledge as ‘satisfying’ or worse [7]. In order to increase the acceptance of risk assessments, this study proposes a PFMEA template. In particular, the template was compiled for a general external beam radiation therapy (EBRT) process.

Various ways of performing FMEA have been described in literature to suit the needs of different industries. Here, we used the PFMEA approach as described in the FMEA handbook of the Automotive Industry Action Group (AIAG) [8] which has, to the best of our knowledge, not yet been applied to radiotherapy. This approach brings about a crucial innovation in failure mode prioritization, namely the substitution of the traditional risk priority number (RPN) with the action priority (AIAG AP). Both prioritization methods utilize the severity *S* of the failure effect, the occurrence *O* of the failure causes and the detection *D* of the failure causes or failure modes. Whereas the RPN is the simple product of *S*, *O*, and *D*, the AIAG AP is a three-dimensional look-up table that evaluates these parameters individually. This way the AIAG AP gives more weight to severity first, then occurrence, then detection [8]. For each of the 1000 possible *S-O-D* combinations (given 10 steps per parameter), individual action levels can be looked up. In total, there are three different action levels—high (H), medium (M), and low (L)—which imply whether actions must, should^1^, or can be implemented, respectively [8]. Its main intent is failure prevention, as measures reducing the severity or the occurrence lead to a greater reduction of the AP. On the other hand, RPNs give equal weight to *S*, *O*, and *D* and therefore, there is no preferred mitigation strategy (reducing severity and increasing detection are of the same value).

Since the AIAG AP table was designed to work with the *S*-*O*-*D* rating systems provided in the FMEA handbook, it should be reviewed before using different rating systems [8]. An example rating system which is used in our department is given in Table 1. Instead of reviewing all 1000 cells of the AIAG AP table, we propose another method that mimics the AIAG AP but which can more easily be adapted to our or any other rating system. In the following, this method is called AP for radiation oncology (RO AP).

**Table 1:** Ten-step rating system used in our department for classifying failure modes in terms of severity of consequences (S), likelihood of occurrence (O) and difficulty of detection (D). Toxicities rated between S = 5 and S = 10 correspond to the five grades of the Common Terminology Criteria for Adverse Events (CTCAE) [9].

Rating	Severity S	Occurrence O	Detectability D
10	Premature death	Many times a day	Almost impossible (between 50% and 100%)
9	Life-threatening	Several times a day	Very remote (50%)
8	Severe toxicity or tumor underdosage (> 20%)	Each day	Remote (20%)
7	Moderate toxicity or tumor underdosage (10%-20%)	Several times a week	Very low (10%)
6	Mild toxicity or tumor underdosage (5%-10%)	Once a week	Low (5%)
5	Side effects requiring intervention	Several times a month	Moderate (2%)
4	Mild side effects	Once a month	Moderately high (1%)
3	Disruption	Several times a year	High (0.5%)
2	Inconvenience	Once a year	Very high (0.2%)
1	No harm	Virtually never (failure causes not possible)	Almost certain (0.01%)

The purpose of this study was thus twofold: Firstly, an EBRT-PFMEA template was created that can be used as the basis for risk assessments across different radiation oncology institutions. The second purpose was to introduce the RO AP as an alternative to the AIAG AP, for example, to rate the failure modes of the EBRT-PFMEA template. The RO AP was benchmarked against the AIAG AP and also against the RPN to investigate the alleged superiority of the AP over the RPN. This benchmark employed Monte Carlo simulations. These two purposes were followed independently, i.e., the template did not affect the simulations and the RO AP was not used to rate the failure modes of the template.

## 2 Methods and materials

### 2.1 EBRT-PFMEA template

A Microsoft Excel PFMEA work sheet was used to compile the template PFMEA (Form F in the FMEA handbook [8]). The AIAG approach involved seven steps:

1. Planning and preparation to set the project scope,
2. structure analysis to describe the process flow,
3. function analysis to describe the functions of the structures,
4. failure analysis to deduce failure chains consisting of a failure effect, failure mode, and failure cause,
5. risk analysis to evaluate failure chains considering prevention or detection controls,
6. optimization to identify further controls,
7. results documentation to communicate the conclusions of the PFMEA [8].

We conducted steps 2 to 6 as described hereafter.

#### 2.1.1 Structure analysis

During the structure analysis, the EBRT process was described by breaking it down into so-called process steps, process items, and process work elements [8]. The process step is the focus of the analysis [8] and, here, referred to an operation that the patient or the patient’s file passes through. The process item is the result of the process step and process work elements are the needed resources. Here, these were identified by means of the 4M (man, machine, method, and material) as commonly known from the Ishikawa approach [10].

Let physical treatment planning be an exemplary process step. A DICOM RT Plan file can be treated as the physical result and therefore as a process item. To conduct the planning, one would typically need dosimetrists/physicists (man), computers (machine), dose algorithms (method), and beam basic data (material).

We used the general EBRT process published in the AAPM’s consensus recommendations for incident learning systems [11] as the basis for the structure analysis. Missing information considered standard of care in today’s practice have been added to the best of our knowledge.

#### 2.1.2 Function analysis

During the function analysis, functions were given to the process structures identified before. Functions describe intents and/or requirements and form the functional relationships between the process structures (what is being done and how is it achieved). The requirements are quantifiable features and can be measured or judged.

Using the physical planning example from above, one typical function is the dosimetrist selecting the correct beam data, dose algorithm, clinical goals, and dose constraints. Another function is the computer calculating the monitor units. Requirements for the plan, for example, are dose-volume-histogram (DVH) parameters.

#### 2.1.3 Failure analysis

In the subsequent failure analysis, all previously identified functions of the process items, process steps, and process work elements were negated and thus became the failure effects, failure modes, and failure causes, respectively. Here, failure effects were described on the process item level as well as the patient level.

In the above example, if the dosimetrist selects the wrong dose algorithm (cause), then doses to organs-at-risk might be underestimated but, in reality, exceed limits (mode). In effect, DVH parameters might actually be not fulfilled (process item level) which might result in toxicities (patient level).

Negating all process step functions in all possible ways would yield all potential failure modes. However, only those associated with failure effects affecting the patient’s safety were relevant here. To evaluate *what is potentially wrong with the process*, we assumed that all process steps are carried out, meaning that errors due to omission were not considered. In an attempt to identify this subset of failure modes we incorporated an extensive literature study. On PubMed.gov (National Library of Medicine, Bethesda, MD, U.S.), the following keywords were used: ‘FMEA’, ‘risk analysis’, ‘risk assessment’ and ‘radiation therapy’. 126 publications were found at the time of this study, of which 43 were relevant for linac-based EBRT. Actual information on failure modes were provided in 33 publications: [2], [3], [4], [5], [6], [12], [13], [14], [15], [16], [17], [18], [19], [20], [21], [22], [23], [24], [25], [26], [27], [28], [29], [30], [31], [32], [33], [34], [35], [36], [37], [38], [39]. To keep the EBRT-PFMEA template manageable as a practical starting point, up to ten of the highest ranked failure modes per study were reviewed. Thereafter, incident reports were analyzed to deduce failure modes that actually occurred. To this end, the WHO’s *Radiotherapy Risk Profile* was used that provided a general list of 48 high-impact errors [40]. Furthermore, 16 ASTRO’s/AAPM’s RO-ILS quarterly reports were used that briefly summarized 7968 incidents and reviewed 69 cases of recurring themes [41], [42], [43], [44], [45], [46], [47], [48], [49], [50], [51], [52], [53], [54], [55], [56]. Lastly, two annual reports of the German reporting and information system for significant events related to radiation exposures in medicine [57], [58] were considered that summarized 100 events relevant for RT. Failure modes retrieved from the literature survey have been rephrased to match the negation of a process step function. The same principle was applied to failure causes and failure effects. Our literature-informed approach directed the focus of the study on high-risk as well as observed failure modes.

#### 2.1.4 Risk analysis

In the subsequent risk analysis, prevention and detection controls that were already integrated into the process were identified. This was done by investigating which functions further upstream or downstream in the process were potentially capable of preventing or detecting failure causes or failure modes.

Ratings for *S*, *O*, and *D* were not given as they will be, due to being rather subjective, different for each institution and risk assessment team.

#### 2.1.5 Optimization

In the optimization section, further potential prevention and detection controls were proposed if the process itself had no adequate control strategies. They were adopted from the literature survey when given.

### 2.2 Radiation oncology action priority

#### 2.2.1 Definition

The RO AP was defined by means of a weighted sum:

$$RO\;AP\left(y\right)=\left\{\begin{array}{c} \mathrm{very}\;\mathrm{high}\;\mathrm{if}\;y\geq y_{\mathrm{vh}}\\ \mathrm{high}\;\mathrm{if}\;y\geq y_{h}\\ \mathrm{medium}\;\mathrm{if}\;y\geq y_{m}\\ \mathrm{low}\;\mathrm{if}\;y<y_{m}\;or\;S=1\;or\;O=1 \end{array}\right)$$

with $y=w_{S}S+w_{O}O+w_{D}D$, and *w_S_*, *w_O_*, and *w_D_* being weighting factors that satisfied $w_{S}\geq w_{O}\geq w_{D}$ as well as $w_{S}+w_{O}+w_{D}=3$. This way, the weighted sum y never exceeded 30, which is the maximum number one can achieve using a ten-step rating system. A fourth action level, very high (VH), was introduced to distinguish actions that must be implemented immediately.

#### 2.2.2 Benchmark

Monte Carlo simulations were performed in order to compare the number of needed actions as required by the AIAG AP, RO AP, and the RPN. We also compared whether the three methods would lead to comparable optimized states. This comparison required establishing the near-same conditions for the RO AP and RPN which is described hereafter.

##### 2.2.2.1 Conditioning the RO AP

The threshold values *y_VH_*, *y_H_*, and *y_M_* had to be determined first in order to create RO AP tables that have nearly the same number of high, medium and low combinations as the AIAG AP table. Of the 1000 *S-O-D* combinations in the AIAG AP table, 318, 214 and 468 combinations refer to H, M, and L, respectively. We identified general threshold values iteratively by using 10,000 random sets of weighting factors {*w_S_, w_O_, w_D_*}_1_, …, {*w_S_, w_O_, w_D_*}_10,000_ to generate 10,000 random RO AP tables. The threshold values were adjusted until nearly the same numbers of H, M, and L combinations were obtained on average. For that purpose, very high was treated the same as high.

##### 2.2.2.2 Conditioning the RPN

To be able to compare the RPN with the AIAG AP, RPN action levels must be established that mimic the purpose of the AP action levels. We referred to the RPN action levels provided by the BFS, DEGRO, DGMP, and DGN [59]. To set up the analogy, RPN values greater than 125 were treated the same as H ratings, and RPN values between 125 and 30 were treated as M ratings. However, these values were derived from a different *S-O-D* rating system as the one used in the FMEA handbook. Therefore, *S-O-D* numbers of the FMEA handbook were mapped to the respective criteria of the joint recommendations. For example, the criterion ‘more than once per day’ is *O* = 9 or *O* = 10 in the joint recommendations and *O* = *7* in the FMEA handbook. After the mapping process (see Supplementary Materials for details), the new RPN action levels were 110 (H) and 27 (M).

##### 2.2.2.3 Monte Carlo simulation

**Figure 1:**
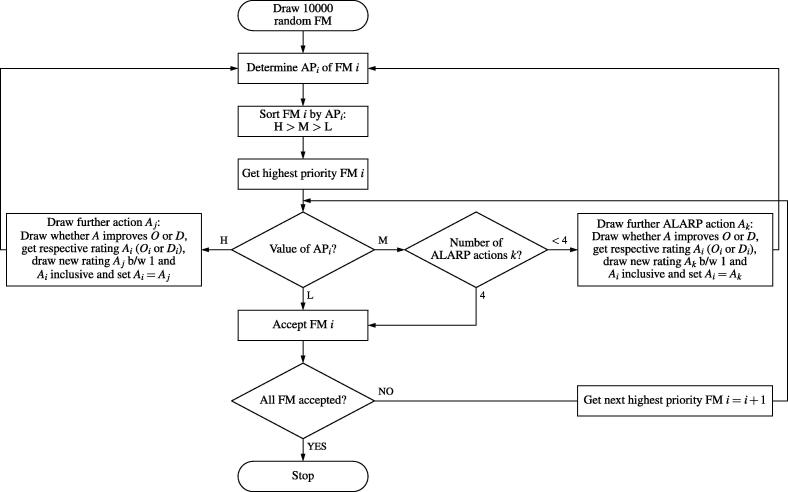
Algorithm for optimizing a set of failure modes (FM) by drawing random corrective actions. This flowchart illustrates the optimization using the AIAG AP as prioritizing method. AP: action priority, H: high, M: medium, L: low, S: severity, O: occurrence, D: detection, ALARP: as low as reasonably practicable.

The Monte Carlo simulations were performed for the AIAG AP as illustrated in Figure 1. The RO AP and RPN simulations were processed analogously with their respective action levels (see 2.2.2.1, 2.2.2.2).

**Figure 2:**
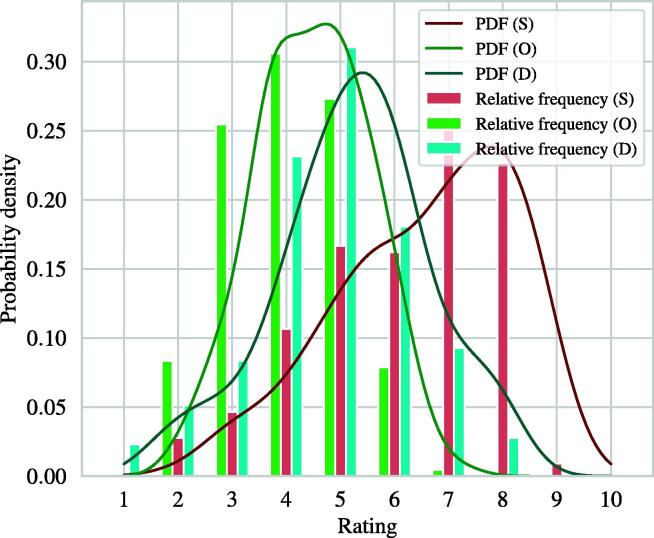
Relative frequencies and estimated probability density functions (PDF) of the 216 S-O-D failure mode ratings given in the AAPM TG-100 report [60].

The start of the simulations was to draw 10,000 random failure mode ratings, i.e., *S-O-D* combinations. To obtain more realistic ratings, the *S-O-D* combinations were drawn from the probability density functions (PDFs) shown in Figure 2. These PDFs were calculated from 216 *S-O-D* ratings provided by AAPM TG-100 [60] using a Gaussian kernel density estimator and Silverman’s rule of thumb [61] as bandwidth estimator.

In the AIAG AP track, the AIAG APs of the 10,000 random *S-O-D* ratings were deduced. This determined the sequence of optimization. Then, during the actual optimization, random prevention and detection ‘actions’ were drawn uniformly. This means that either the *O* or *D* rating was randomly improved but never both ratings at once. Actions were continuously drawn until the optimized AIAG AP was either M or L. In case of an (optimized) M rating, (further) actions were drawn until a maximum number of four (this number was considered as sufficient defense in depth by IAEA-TECDOC-1685 [62]). Failure mode ratings were considered acceptable once there was either sufficient defense in depth or an L rating.

In the RO AP track, the simulation was repeatedly conducted for 40 random sets of weighting factors {*w_S_, w_O_, w_D_*}_1_, …, {*w_S_, w_O_, w_D_*}_40_. This would reveal whether there was a relationship between these and the number of needed actions. Moreover, if a set of weighting factors existed that could replicate the AIAG AP optimization, it could then be determined by interpolation.

Once all failure modes were accepted in each track, the average numbers of actions per failure mode could be determined. Additionally, we quantified the achieved optimized states by means of the residual total criticality number ΣSO as a surrogate. The criticality number SO is the product of severity and occurrence and directs the focus on reducing the occurrence of failure modes [63].

Finally, we parametrized the RO AP with the found sets of threshold values and weighting factors to reproduce the AIAG AP. Then, the median RPN values of the four action levels were determined and compared, using the Kruskal-Wallis *H* test, a non-parametric analysis of variance.

#### 2.2.3 Adaption

The general threshold values found in section 2.2.2.1 were substituted with appropriate threshold values to calibrate the RO AP for the *S-O-D* rating system given in Table 1. These were chosen manually so that the following intentions were satisfied: If failure modes resulted in mild side effects at worst (*S* ≤ 4) and occurred once a month or less (*O* ≤ 4), then an acceptable state (L) should easily be achievable because the benefit of the treatment far surpasses the risks. If S ≤ 6 (up to mild toxicity), then depending on the occurrence, actions should or must be taken (M/H) because toxicities, in general, should be avoided. If *S* ≥ 8 (up to premature death), actions must immediately be taken (VH) because the benefit of the treatment is greatly reduced.

## 3 Results

### 3.1 EBRT-PFMEA Template

On the basis of the AIAG PFMEA approach, an EBRT-PFMA template with 9 process items, 33 process steps, and 112 process functions could be compiled. The template is given to the reader in full detail via the Supplementary Materials. Here, an excerpt from the template, showing a subset of the structure and function analysis, is shown in Table 2.

**Table 2:** Overview of the process items, steps and functions identified by means of the structure and function analysis. Up to three associated process steps and functions are shown. Full details are provided in the Supplementary Materials.

Process Item	Process Step	Process Function
Patient assessment & consultation	Registration	Register patient for initiating treatment process
	Diagnostics	Collect and review (outside) diagnostical data as input for therapeutic goals
	Tumor board review	Recommend therapeutic options
Imaging for RT planning (DICOM studies)	Identification	Check-in patient for imaging
	RT imaging	Accurate and reproducible imaging of patient's anatomy/geometry for planning
	Other imaging	Support definition of correct target delineation
Medical planning (DICOM structure set)	Pre-planning	Prepare imaging studies for medical planning
	Plan reconstruction	Reconstruct sum of previous RTs isodoses on current planning CT
	Contouring	Render geometrically accurate 3D model of patient targets and organs-at-risk reflecting therapeutic goals
Physical planning (DICOM plan)	Physical planning	Develop appropriate patient specific treatment plan
	Physical planning review	Evaluate quality of treatment plan to achieve therapeutic goals
Treatment preparation	Treatment preparation	Prepare treatment plan for treatment delivery
	Documentation	Document and archive treatment data (DICOM planning CT, structure sets and treatment plans)
Pre-treatment verification	Verification	Review of correct treatment parameters
		Verification of predicted dose calculation
Treatment delivery	Identification	Check-in patient for daily treatment
	Patient preparation	Preparation of patient and treatment unit for treatment delivery
	Positioning / immobilization	Reproducible positioning and immobilization of patient (reproduce simulation scenario)
On-treatment quality management	Documentation	Document delivered radiation beam to patient's tumor site
	Treatment review	Review validity of treatment and adapt to occurring changes in a timely manner
	Treatment change control	Changes to treatment are documented and implemented
Post-treatment completion	Treatment summary	Record/document/archive treatment data (plans, images, etc.)
	Follow up	Monitor treatment outcome

During the failure analysis, over 1400 failure modes could be gathered through the literature survey and 75 distinct, high-risk failure chains could be identified. 70.7% failure modes were preventable or detectable by the process itself which indicated the already high level of inherent safety of the general process map. In addition, 92 prevention controls and 70 detection controls were identified that could be implemented by users of the template and were not already part of the process. Among the prevention controls, standardized policies, documentation and communication strategies as well as key performance indicators (KPI) were most dominant. Checklists and the four-eyes-principle were a common theme for detecting failure modes or causes.

**Figure 3:**
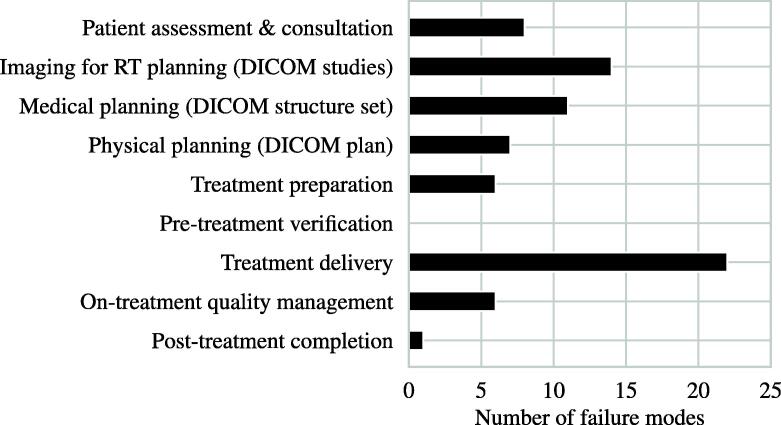
Process items and associated number of identified failure modes of the radiotherapy chain.

**Figure 4:**
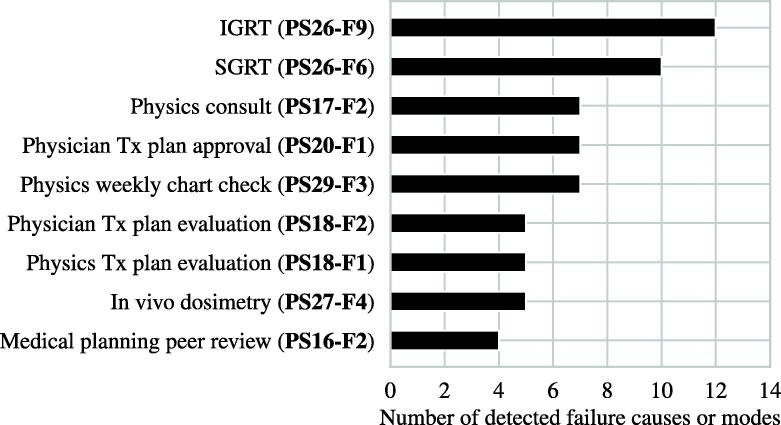
Most effective process functions detecting at least four of the 75 identified failure causes or modes. IGRT: Image guided radiation therapy, SGRT: surface guided radiation therapy, Tx: treatment.

Figure 3 shows the number of failure modes in each process step. With 22 (29.3%) failure modes, treatment delivery was most prone to error. Treatment planning, i. e., medical and physical planning, followed with 18 (24%) failure modes in total. 14 (18.7%) failure modes were identified for primary RT imaging. In Figure 4, process functions acting as detection controls are listed. Being able to detect 16% failure modes, image guided verification was the most effective detection control. Followed by surface guided radiotherapy and physics (treatment planning) consult, 13.9% and 9.7% of identified failure modes were potentially detectable, respectively.

### 3.2 Radiation Oncology Action Priority

#### 3.2.1 Benchmark

Based on random sets of weighting factors, we found *y_VH_* = 24.0, *y_H_* = 19.2 and *y_M_* = 15.5 to be suitable and general threshold values that yielded 318 VH and H, 212 M and 470 L combinations on average.

**Figure 5:**
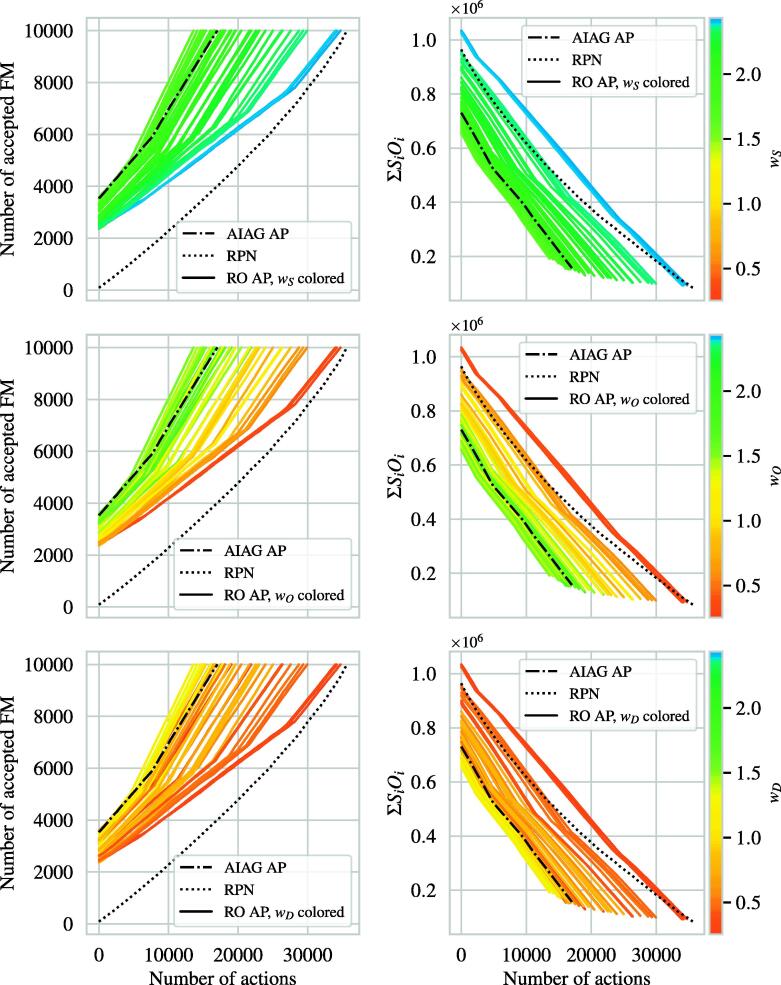
A set of 10,000 random failure modes was optimized by means of the AIAG AP, RO AP and RPN and drawing random corrective actions until all failure modes were considered acceptable (left). On the right, the residual total criticality number is shown. The colored lines indicate the different random weighting factors used for the RO AP. FM: failure mode, AP: action priority, RPN: risk priority number.

Figure 5 shows the optimization processes obtained through the Monte Carlo simulations. There are two crucial observations: Firstly, the RO AP could theoretically replicate the AIAG AP with regards to the number of necessary actions (left and right). The associated set of weighting factors was found to be *w_S_* = 1.34, *w_O_* = 1.20, and *w_D_* = 0.46. Then, the RO AP would require 1.7 actions per failure mode, as did the AIAG AP. The greater *w_S_*, the more actions would be necessary because the algorithm only drew prevention (*O*) and detection (*D*) controls.

Secondly, the RPN optimization stopped at a value of the residual total criticality number ΣSO which was approximately 47% lower than the value obtained through the AIAG AP (right). This indicated that the risk acceptance of the BFS, DEGRO, DGMP, and DGN [59] was more restrictive than the AIAG’s risk acceptance. This inequality increased the required number of actions by 2.1 to 3.6 actions per failure mode. Another consequence was the apparently slower optimization. The average slope for the AIAG AP was –33.7 ΣSO per action, whereas it was –24.7 ΣSO per action for the RPN.

**Figure 6:**
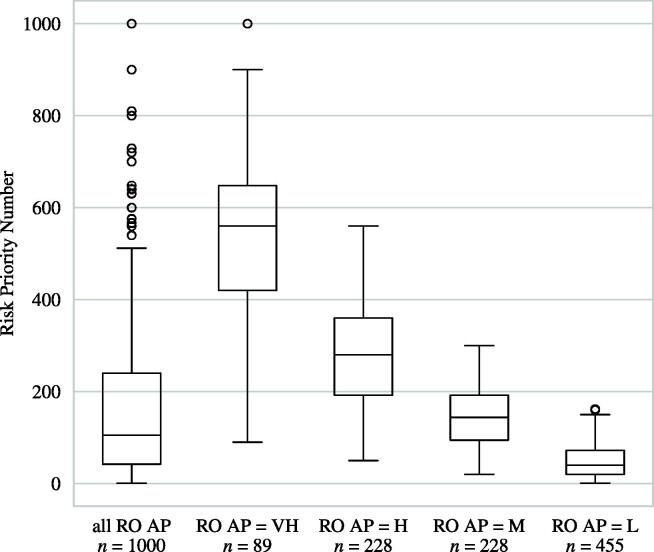
The RO AP with four action levels subdivides the RPN distribution of all 1000 possible S-O-D combinations into significantly different groups leading to a superior optimization process. For the above classification, w_S_ = 1.34, w_O_ = 1.20, w_D_ = 0.46, y_VH_ = 24.0, y_H_ = 19.2 and y_M_ = 15.5 were used to determine the RO AP levels.

Figure 6 shows box plots of the total RPN distribution (leftmost) as well as RPN sub-distributions classified as RO AP action levels. Using the Kruskal-Wallis *H* test, it could be shown that the medians of all RPN sub-distributions were significantly different from each other (*p* < 0.00001).

#### 3.2.2 Adaption

We could identify an RO AP table reflecting the intentions described in section 2.2.3 using the threshold values *y_VH_* = 23.0, *y_H_* = 18.0, and *y_M_* = 14.0 (see Supplementary Materials). This RO AP parametrization had 395 (+24.2%) VH and H, 229 (+8.0%) M, and 376 (-20.0%) L combinations and required around 2.4 corrective actions per failure mode (+41.4%). Again, the Kruskal-Wallis *H* test showed significantly different RPN medians of all groups (*p* ≈ 0).

## 4 Discussion

### 4.1 EBRT-PFMEA Template

In this study, we compiled a practical EBRT-PFMEA template. The advantages of templates are multifold: For example, templates may serve as input for identifying failure modes that have been overlooked in an FMEA started from scratch [2]. In automotive industry, templates are already a common and recommended practice that reduces resources and accumulates past experiences and knowledge from lessons learnt [8]. Templates could also contribute to standardizing the quality of risk assessments across institutions [64]. If mundane causes such as little knowledge or lack of staff or time lower risk assessment efforts, then templates could improve and speed up these efforts. Certainly, little knowledge was true for our department in the phase just after risk assessments were made obligatory. However, as radiation oncology departments gain experience and future medical physics experts in the EU should acquire risk management skills [65], these causes might diminish over time.

In this study, the AIAG PFMEA approach has been applied which differs from other established approaches, such as the ones recommended by AAPM TG-100 [60] or by the BFS, DEGRO, DGMP, and DGN [59]. All three approaches have in common that the process is mapped out, however, the AIAG approach goes beyond that, inter alia, by performing a structure and function analysis. These steps are immensely crucial because they establish what is being done by whom and what needs to be achieved. Defining these structures and functions makes deducing failure modes a purely logical task. This is because the failure modes are simply the negated process step functions. For instance, a function executed incorrectly, too early, too late, in excess or insufficiently, etc. will represent a failure mode. In the other two approaches, identifying failure modes was immediately being done after the process had been mapped out. There, identifying failure modes rather resembled a brainstorming exercise (what could possibly go wrong?). Because of that, the quality of the identified failure modes will necessarily be dependent of the experience of the analysts (obtained, for instance, through past undesirable events).

Even though the literature survey provided over 1400 failure modes, we could not include all of them due to practical reasons. Repeated and specialized failure modes have been combined and abstracted. All failure modes that could be eliminated due to the EBRT process design used here and those representing errors due to omission (e. g., ‘peer review not performed’, ‘IGRT not performed’, etc.) have been sorted out. Obviously, patient safety is compromised if crucial functions are not performed. For instance, vertebral bodies were mixed-up in the past because the patient position had not been verified [49]. However, PFMEA is a tool for analyzing *what is wrong with the process* [66], thereby identifying safety-improving process functions. To make sure these functions are not omitted, other tools such as checklists should be employed. The process of compiling FMEA-based checklists is illustrated in detail in AAPM TG-275 [38].

According to Figure 3, there were no failure modes of the pre-treatment verification process, which was unexpected. However, the pre-treatment verification itself serves as an extensive detection method of failure modes that occurred in previous steps. For instance, if an incorrect dose distribution was calculated during physical planning, this should be detected during verification but the respective failure mode is belonging to the planning process. Actual failure modes of the verification (e.g., incorrect execution of measurements) were not included as the patient safety was not considered to be directly compromised through these modes.

### 4.2 Usage of the EBRT-PFMEA Template

In a first step, the reader modifies the template provided in the Supplementary Materials in order to create a local template valid for their institution. Because of that, our EBRT-PFMEA template was kept rather general so that differences to the reader’s specific EBRT process can be quickly identified and either be added or removed. The provided process structures and functions should be adapted to the institution’s situation first. Some failure modes might be eliminated or new site-specific failure modes emerge as a consequence. We do not recommend removing crucial process functions since these ensure a high level of inherent safety, as can be seen in Figure 4. In a second step, the reader’s local template is then used for the actual risk assessment of, e.g., the launch of a new treatment unit or a new treatment technique.

### 4.3 Limitations of the EBRT-PFMEA Template

The EBRT-PFMEA template has not yet been clinically validated. Moreover, we restricted the literature review to the ten highest ranked failure modes per article which all have been ranked by the RPN. Since RPNs are ambiguous in terms of level of risk [67], some high-risk failure modes might have not appeared in the top ten of each article. This could result in an incomplete template.

### 4.4 Radiation Oncology Action Priority

In this study, we also investigated how the novel action priority concept could be transferred to radiation oncology. The dominating concept in radiation oncology is, however, not the AIAG AP but the RPN, due to the task report TG-100 [60], the joint recommendations of the BFS, DEGRO, DGMP, and DGN [59] and many other publications. For the AP to be accepted, a proof showing its superiority over the RPN seemed necessary, while at the same time it should easily be adaptable to any rating system, i.e., without the need for reviewing all 1000 cells.

In other studies, it has already been shown that the AIAG AP increased the chances of reaching a team consensus (i. e., the same action level) because it only offers three action levels [68]. Using a ten-step rating system, the variance can be as high as 7 steps between individuals, so in the case of the RPN, there could be an uncertainty factor of up to 12 [69]. Therefore, the chances of reaching the same consensus are lower. Furthermore, Barsalou recently compared high APs versus RPN values greater than 100 and showed a significant reduction in the number of corrective actions [70].

Here, we propose the RO AP, a transparent method for calibrating the AP table for any rating system, as an alternative to the fixed AIAG AP table. Building on Braband’s sum rule for the RPN [67], we added weighting factors and threshold values to their formula. We could show that the RO AP and AIAG AP were practically equivalent in terms of optimization (Figure 5).

Moreover, we could verify that the two AP approaches indeed reduced the number of corrective actions compared to the RPN, underlining Barsalou’s findings [70]. However, results of this kind are rather arbitrary because AP and RPN action levels themselves are arbitrary [71]. For example, Noel et al. [33] considered RPN ≥ 200 as critical, implying presumably less corrective actions. We could observe that adjusting the RO AP threshold values to our needs increased the number of actions per failure mode from 1.7 to 2.4. Thus, comparing the AP and the RPN concepts should always be done with caution.

As can be seen in Figure 6, RPNs between 90 and 162 could be any of the four RO AP action levels. As reference, about 38.9% TG-100 failure modes lie in this interval. Our results imply that a considerable amount of failure modes in radiation oncology could be optimized in a more effective way by utilizing the RO AP.

### 4.5 Limitations of the RO AP

The simulation simplified reality. It was assumed that there are unlimited resources so that all failure modes could be optimized. In reality, teams might not find suitable measures or justify that current measures are already sufficient, for example because risks are considered to be already as low as reasonably achievable (ALARA). Furthermore, the failure-prevention intent of the AP was neglected as severity reducing actions were not simulated, even though they would reduce the AP most drastically. In reality, the actual number of actions per failure mode will likely be lower due to aforementioned reasons.

## 5 Conclusion

A practical necessity for a general FMEA template arises due to legal obligations in the EU to perform risk assessments for unintentional exposures to radiation [72]. Practitioners lacking knowledge in risk management procedures are furnished an adjustable model that helps them in designing a safe process map, identifying failure modes and monitoring effects of control mechanisms over time. In sharing the template, it could become a standardized starting point for all EBRT-based processes. In a collective approach to increase patient safety, we hope to encourage other authors with this initiative to provide FMEA templates for, e. g., brachytherapy or other branches of radiotherapy.

The RO AP can reproduce an optimization process similar to the AIAG AP. However, the RO AP has the advantage that it founds on a rather simple weighted sum which makes the action levels of all 1000 *S-O-D* combinations transparent. In addition, the RO AP table can easily be adapted to any rating system. This replaces the necessity to inspect all cells of the AP table individually when modifying the rating system.

## CRediT authorship contribution statement

**Dominik Kornek:** Conceptualization, Writing - original draft, Visualization, Investigation, Validation, Formal analysis, Methodology. **Christoph Bert:** Funding acquisition, Writing - review & editing, Validation, Methodology, Supervision.

## Declaration of competing interest

The authors declare the following financial interests/personal relationships which may be considered as potential competing interests: This project was funded by the Bavarian Ministry of Economic Affairs, Regional Development and Energy (grant number 07 03/686 68/288/21/7/22/8/23/9/24, 07 03/686 68/287/21/4/22/5/23/6/24) and was performed in collaboration with IBA Dosimetry GmbH (Schwarzenbruck, Germany). IBA Dosimetry GmbH had no involvement with the work reported in this paper.

## Footnotes

1 The reduced risk level should be as low as reasonably practicable (ALARP) to be generally acceptable.
