# Supplementary material for: Process failure mode and effects analysis for external beam radiotherapy: Introducing a literature-based template and a novel action priority
Source: Z Med Phys. 2024 Feb 29;34(3):358–70. doi: 10.1016/j.zemedi.2024.02.002 (PMC11384953; doi:10.1016/j.zemedi.2024.02.002)
Supplement: Supplementary Data 1 [file mmc1.docx]

# Supplementary Materials

Table 1: Radiation oncology action priority (RO AP) table with four action levels using y_VH_ = 23.0, y_H_ = 18.0, y_M_ = 14.0, w_S_ = 1.34, w_O_ = 1.20, and w_D_ = 0.46. Severity (S), occurrence (O) and detectability (D) criteria are given in Table 1 of the main document. VH: very high priority, H: high priority, M: medium priority, L: low priority.

| S | O | | | | | | | | | | D |  | S | O | | | | | | | | | | D |
| --- | --- | --- | --- | --- | --- | --- | --- | --- | --- | --- | --- | --- | --- | --- | --- | --- | --- | --- | --- | --- | --- | --- | --- | --- |
|  | 10 | 9 | 8 | 7 | 6 | 5 | 4 | 3 | 2 | 1 |  |  |  | 10 | 9 | 8 | 7 | 6 | 5 | 4 | 3 | 2 | 1 |  |
| 10 | VH | VH | VH | VH | VH | VH | H | H | H | L | 10 |  | 5 | VH | H | H | H | H | M | M | M | L | L | 10 |
|  | VH | VH | VH | VH | VH | VH | H | H | H | L | 9 |  |  | H | H | H | H | H | M | M | M | L | L | 9 |
|  | VH | VH | VH | VH | VH | VH | H | H | H | L | 8 |  |  | H | H | H | H | M | M | M | L | L | L | 8 |
|  | VH | VH | VH | VH | VH | H | H | H | H | L | 7 |  |  | H | H | H | H | M | M | M | L | L | L | 7 |
|  | VH | VH | VH | VH | VH | H | H | H | H | L | 6 |  |  | H | H | H | M | M | M | M | L | L | L | 6 |
|  | VH | VH | VH | VH | H | H | H | H | H | L | 5 |  |  | H | H | H | M | M | M | L | L | L | L | 5 |
|  | VH | VH | VH | VH | H | H | H | H | M | L | 4 |  |  | H | H | H | M | M | M | L | L | L | L | 4 |
|  | VH | VH | VH | VH | H | H | H | H | M | L | 3 |  |  | H | H | M | M | M | M | L | L | L | L | 3 |
|  | VH | VH | VH | H | H | H | H | M | M | L | 2 |  |  | H | H | M | M | M | L | L | L | L | L | 2 |
|  | VH | VH | VH | H | H | H | H | M | M | L | 1 |  |  | H | M | M | M | M | L | L | L | L | L | 1 |
| 9 | VH | VH | VH | VH | VH | H | H | H | H | L | 10 |  | 4 | H | H | H | H | M | M | M | L | L | L | 10 |
|  | VH | VH | VH | VH | VH | H | H | H | H | L | 9 |  |  | H | H | H | M | M | M | M | L | L | L | 9 |
|  | VH | VH | VH | VH | H | H | H | H | H | L | 8 |  |  | H | H | H | M | M | M | L | L | L | L | 8 |
|  | VH | VH | VH | VH | H | H | H | H | M | L | 7 |  |  | H | H | H | M | M | M | L | L | L | L | 7 |
|  | VH | VH | VH | VH | H | H | H | H | M | L | 6 |  |  | H | H | M | M | M | M | L | L | L | L | 6 |
|  | VH | VH | VH | H | H | H | H | M | M | L | 5 |  |  | H | H | M | M | M | L | L | L | L | L | 5 |
|  | VH | VH | VH | H | H | H | H | M | M | L | 4 |  |  | H | H | M | M | M | L | L | L | L | L | 4 |
|  | VH | VH | VH | H | H | H | H | M | M | L | 3 |  |  | H | M | M | M | L | L | L | L | L | L | 3 |
|  | VH | VH | H | H | H | H | M | M | M | L | 2 |  |  | H | M | M | M | L | L | L | L | L | L | 2 |
|  | VH | VH | H | H | H | H | M | M | M | L | 1 |  |  | M | M | M | M | L | L | L | L | L | L | 1 |
| 8 | VH | VH | VH | VH | H | H | H | H | M | L | 10 |  | 3 | H | H | H | M | M | M | L | L | L | L | 10 |
|  | VH | VH | VH | VH | H | H | H | H | M | L | 9 |  |  | H | H | M | M | M | M | L | L | L | L | 9 |
|  | VH | VH | VH | H | H | H | H | H | M | L | 8 |  |  | H | H | M | M | M | L | L | L | L | L | 8 |
|  | VH | VH | VH | H | H | H | H | M | M | L | 7 |  |  | H | H | M | M | M | L | L | L | L | L | 7 |
|  | VH | VH | VH | H | H | H | H | M | M | L | 6 |  |  | H | M | M | M | L | L | L | L | L | L | 6 |
|  | VH | VH | H | H | H | H | M | M | M | L | 5 |  |  | H | M | M | M | L | L | L | L | L | L | 5 |
|  | VH | VH | H | H | H | H | M | M | M | L | 4 |  |  | M | M | M | M | L | L | L | L | L | L | 4 |
|  | VH | H | H | H | H | H | M | M | M | L | 3 |  |  | M | M | M | L | L | L | L | L | L | L | 3 |
|  | VH | H | H | H | H | M | M | M | M | L | 2 |  |  | M | M | M | L | L | L | L | L | L | L | 2 |
|  | VH | H | H | H | H | M | M | M | L | L | 1 |  |  | M | M | M | L | L | L | L | L | L | L | 1 |
| 7 | VH | VH | VH | H | H | H | H | M | M | L | 10 |  | 2 | H | H | M | M | M | L | L | L | L | L | 10 |
|  | VH | VH | VH | H | H | H | H | M | M | L | 9 |  |  | H | M | M | M | M | L | L | L | L | L | 9 |
|  | VH | VH | H | H | H | H | M | M | M | L | 8 |  |  | H | M | M | M | L | L | L | L | L | L | 8 |
|  | VH | VH | H | H | H | H | M | M | M | L | 7 |  |  | M | M | M | M | L | L | L | L | L | L | 7 |
|  | VH | H | H | H | H | H | M | M | M | L | 6 |  |  | M | M | M | L | L | L | L | L | L | L | 6 |
|  | VH | H | H | H | H | M | M | M | M | L | 5 |  |  | M | M | M | L | L | L | L | L | L | L | 5 |
|  | VH | H | H | H | H | M | M | M | L | L | 4 |  |  | M | M | M | L | L | L | L | L | L | L | 4 |
|  | H | H | H | H | M | M | M | M | L | L | 3 |  |  | M | M | L | L | L | L | L | L | L | L | 3 |
|  | H | H | H | H | M | M | M | L | L | L | 2 |  |  | M | M | L | L | L | L | L | L | L | L | 2 |
|  | H | H | H | H | M | M | M | L | L | L | 1 |  |  | M | L | L | L | L | L | L | L | L | L | 1 |
| 6 | VH | VH | H | H | H | H | M | M | M | L | 10 |  | 1 | L | L | L | L | L | L | L | L | L | L | 10 |
|  | VH | H | H | H | H | H | M | M | M | L | 9 |  |  | L | L | L | L | L | L | L | L | L | L | 9 |
|  | VH | H | H | H | H | M | M | M | M | L | 8 |  |  | L | L | L | L | L | L | L | L | L | L | 8 |
|  | VH | H | H | H | H | M | M | M | L | L | 7 |  |  | L | L | L | L | L | L | L | L | L | L | 7 |
|  | H | H | H | H | H | M | M | M | L | L | 6 |  |  | L | L | L | L | L | L | L | L | L | L | 6 |
|  | H | H | H | H | M | M | M | L | L | L | 5 |  |  | L | L | L | L | L | L | L | L | L | L | 5 |
|  | H | H | H | H | M | M | M | L | L | L | 4 |  |  | L | L | L | L | L | L | L | L | L | L | 4 |
|  | H | H | H | M | M | M | M | L | L | L | 3 |  |  | L | L | L | L | L | L | L | L | L | L | 3 |
|  | H | H | H | M | M | M | L | L | L | L | 2 |  |  | L | L | L | L | L | L | L | L | L | L | 2 |
|  | H | H | H | M | M | M | L | L | L | L | 1 |  |  | L | L | L | L | L | L | L | L | L | L | 1 |

Table 2: Mapped ten-step rating systems between the AIAG FMEA handbook [1] and the joint recommendations of the BFS, DEGRO, DGMP, and DGN [2].

| **Severity S** | | | |
| --- | --- | --- | --- |
| **Rating in [1]** | **Criteria given in table C2.1 in [1]** | **Rating in [2]** | **Mapped criteria given table 1 in [2]** |
| 10 | High | 10 | Fatal outcome, immediately or shortly after |
| 9 | High | 9 | Fatal outcome, immediately or shortly after |
| 8 | Moderately high | 8 | Permanent impairment of a bodily function as an inevitable consequence |
| 7 | Moderately high | 7 | Permanent impairment of a bodily function as an inevitable consequence |
| 6 | Moderately low | 6 | Impairment requires, e. g., a continuation of the treatment with unclear consequences |
| 5 | Moderately low | 5 | Impairment requires, e. g., a continuation of the treatment with unclear consequences |
| 4 | Moderately low | 4 | Impairment requires, e. g., a continuation of the treatment with unclear consequences |
| 3 | Low | 3 | Temporary impairment |
| 2 | Low | 2 | Temporary impairment |
| 1 | Very low | 1 | No or negligible effect |
| **Occurrence O** | | | |
| **Rating in [1]** | **Criteria given in table C2.3.2 in [1]** | **Rating in [2]** | **Mapped criteria given Table 1 in [2]** |
| 10 | Every time | - | - |
| 9 | Almost every time | - | - |
| 8 | More than once per shift | 10 | Daily, possibly even multiple times |
| 7 | More than once per day | 9 | Daily, possibly even multiple times |
| 6 | More than once per week | 7-8 | Weekly, possibly even multiple times |
| 5 | More than once per month | 6 | Yearly, possibly even monthly |
| 4 | More than once per year | 5 | Yearly, possibly even monthly |
| 3 | Once per year | 4 | Yearly, possibly even monthly |
| 2 | Less than once per year | 2-3 | Approx. once in 5 years |
| 1 | Never | 1 | Unlikely or only once in 5-30 years |
| **Detectability D** | | | |
| **Rating in [1]** | **Criteria given in table C2.4 in [1]** | **Rating in [2]** | **Mapped criteria given Table 1 in [2]** |
| 10 | Very low | 10 | Very low (less than 85% will be detected) |
| 9 | Very low | 9 | Very low (less than 85% will be detected) |
| 8 | Low | 8 | Low (85% to 95% will be detected) |
| 7 | Low | 7 | Low (85% to 95% will be detected) |
| 6 | Moderate | 4-6 | Moderate (95% to 99% will be detected) |
| 5 | Moderate | 4-6 | Moderate (95% to 99% will be detected) |
| 4 | High | 2-3 | High (99% to 99.8% will be detected) |
| 3 | High | 2-3 | High (99% to 99.8% will be detected) |
| 2 | High | 2-3 | High (99% to 99.8% will be detected) |
| 1 | Very high | 1 | Very high (99.99% will be detected) |

To calculate the respective actions levels for the risk priority number given in [2], the cube roots were computed to determine the average values of *S*, *O*, and *D*. For the first action level, 125 = 5³, we can determine the mapped *S-O-D* values to be 5-4-5.5, which gives RPN = 110. For the second action level, 30 = 3.107³, *S-O-D* map to about 3.107-2.107-4.107, which corresponds to an RPN of approx. 27.

[1] *AIAG & VDA FMEA-Handbook. Design FMEA, Process FMEA, Supplemental FMEA for Monitoring & System Response. First Edition Issued June 2019*. AIAG, VDA, 2019.

[2] "Empfehlungen von BfS, DEGRO, DGMP und DGN zur Risikoanalyse bei therapeutischen Strahlenanwendungen nach Artikel 63 Buchstabe b der EU-Direktive 2013/59/Euratom." [Online]. Available: cdn.dgmp.de/media/document/1507/Empfehlungen-zur-Risikoanalyse-finaleVersion.pdf
